# Development of a test battery to enhance safe return to sports after anterior cruciate ligament reconstruction

**DOI:** 10.1007/s00167-016-4246-3

**Published:** 2016-07-16

**Authors:** Alli Gokeler, Wouter Welling, Stefano Zaffagnini, Romain Seil, Darin Padua

**Affiliations:** 1 Center for Human Movement Sciences, University Medical Center Groningen, University of Groningen, Antonius Deusinglaan 1, 9713 AV Groningen, The Netherlands; 2 Medisch Centrum Zuid, Groningen, The Netherlands; 3 Rizzoli Orthopaedic Institute, University of Bologna, Bologna, Italy; 4 Département de l’Appareil Locomoteur, Centre Hospitalier de Luxemburg, Luxemburg, Luxemburg; 5 Department of Exercise and Sport Science, University of North Carolina at Chapel Hill, Chapel Hill, NC USA

**Keywords:** Return to sports, Anterior cruciate ligament reconstruction, Strength, Hop tests, Questionnaires, Second injury, Injury risk

## Abstract

**Purpose:**

There is a lack of consensus regarding the appropriate criteria for releasing patients to return to sports (RTS) after anterior cruciate ligament reconstruction (ACLR). A test battery was developed to support decision-making.

**Methods:**

Twenty-eight patients (22 males and 6 females) with a mean age of 25.4 ± 8.2 years participated and were 6.5 ± 1.0 months post-ACLR. All patients followed the same rehabilitation protocol. The test battery used consisted of the following: isokinetic test, 3 hop tests and the jump-landing task assessed with the LESS. The isokinetic tests and single-leg hop tests were expressed as a LSI (involved limb/uninvolved limb × 100 %). In addition, patients filled out the IKDC and ACL-Return to Sport after Injury (ACL-RSI) scale. RTS criteria to pass were defined as a LSI > 90 % on isokinetic and hop tests, LESS < 5, ACL-RSI > 56 and a IKDC within 15th percentile of healthy subjects.

**Results:**

Two out of 28 patients passed all criteria of the test protocol. The pass criterion for the LESS < 5 was reached by 67.9 % of all patients. For the hop tests, 78.5 % of patients passed LSI > 90 % for SLH, 85.7 % for TLH and 50 % for the SH. For the isokinetic test, 39.3 % of patients passed criteria for LSI peak torque quadriceps at 60°/s, 46.4 % at 180°/s and 42.9 at 300°/s. In total, 35.7 % of the patients passed criterion for the peak torque at 60°/s normalized to BW (>3.0 Nm) for the involved limb. The H/Q ratio at 300°/s > 55 % for females was achieved by 4 out of 6 female patients, and the >62.5 % criterion for males was achieved by 75 %. At 6 months post-ACLR, 85.7 % of the patients passed the IKDC score and 75 % the ACL-RSI score >56 criteria.

**Conclusion:**

The evidence emerging from this study suggests that the majority of patients who are 6 months after ACLR require additional rehabilitation to pass RTS criteria. The RTS battery described in this study may serve as a framework for future studies to implement multivariate models in order to optimize the decision-making regarding RTS after ACLR with the aim to reduce incidence of second ACL injuries.

**Level of evidence:**

III.

## Introduction

Most athletes who wish to continue in sports after an injury to the anterior cruciate ligament (ACL) are advised to undergo ACL reconstruction (ACLR) [28]. However, returning to a high activity level after ACLR is linked with high risk, with reported rates of 23 % in young athletes, to sustain a second ACL injury, either on the ipsilateral or on contralateral side [40]. In a systematic review based on 264 studies included, Barber-Westin and Noyes found that return to sports (RTS) decision was mostly based on subjective non-specific criteria and the majority of studies used time-based decision allowing RTS at 6 months [6]. This clinical approach may need to be revised in light of the high incidence of second ACL injuries.

Recently, a test battery with functional tests for decision-making with regard to RTS following ACLR was published [19]. The test battery included one- and two-legged postural stability tests and jump tasks. These tests are for the most part quantitative in character (e.g. jump height), whereas the quality of movements is not assessed. It has been suggested to incorporate movement analysis to detect asymmetrical movement patterns after ACLR prior to release of the athlete to the high demands of sports [34, 41].

Hence, to support decision-making for RTS, a test battery was developed with physical measures that consisted of isokinetic strength, single hop test for distance (SLH), triple hop for distance (TLH), side hop (SH) and a jump-landing task assessed with the Landing Error Scoring System (LESS) [33]. Two patient questionnaires were added to the RTS test battery. As psychological responses can be attributed to whether patient succeeds or not to RTS, the Anterior Cruciate Ligament-Return to Sport after Injury scale (ACL-RSI) was added [4]. The International Knee Documentation Committee Subjective Knee Form (IKDC) was also added as it has been regarded as an important measure of successful outcome after ACLR [27].

The purpose of this study was to present a test battery that includes both physical and patient-reported measures after ACLR. It was hypothesized that a test battery combining components of physical measures such as strength and single-leg hop tasks and jump-landing tasks in combination with patient-reported outcomes (IKDC and ACL-RSI) would identify deficits in patients following ACLR.

## Materials and methods

Twenty-eight patients, 22 males (mean age 26.2 ± 8.7 years) and 6 females (mean age 22.8 ± 6.4 years), participated. The detailed demographics are presented in Table 1. All subjects were level I–II athletes prior to injury [12]. Inclusion criteria for the patients have been reported previously [14]. An arthroscopic ACLR with anteromedial portal technique was performed on all patients by the same 2 surgeons. Patients completed a rehabilitation programme at the same outpatient physical therapy clinic. In the first 6 weeks after surgery, rehabilitation goals were to reduce inflammation and swelling, restore full knee extension, gait training and neuromuscular training to facilitate quadriceps activity. After approximately 6 weeks, neuromuscular training continued with more advanced drills and muscle strengthening and endurance training were added. At 12 weeks, muscle hypertrophy strengthening was started and running activities and jumping tasks were added. In weeks 24–36, plyometric activities, running/cutting drills, followed by sport-specific agility drills on the field, were initiated. The patients performed the test battery on average 6.5 ± 1.0 months following ACLR. The study protocol was approved by the Medical Ethical Committee (ID 2012.362) of the University of Groningen, and informed consent was obtained from all subjects prior to data collection.

**Table 1:** Descriptive data of included subjects (mean±)

	Age (years)	Weight (kg)	Type graft (*n*)	Time post-surgery (months)	Number of therapy sessions	Lysholm score
All subjects (*n* = 28)	25.5 ± 8.3	78.5 ± 12.7	HT (19), PT (8), AG (1)	6.5 ± 1.0	43.3 ± 13.8	67.6 ± 24.5
Males (*n* = 22)	26.2 ± 8.8	81.6 ± 11.3	HT (14), PT (7), AG (1)	6.5 ± 1.1	43.1 ± 14.7	63.9 ± 25.4
Females (*n* = 6)	22.8 ± 6.4	67.3 ± 11.8	HT (5), PT (1)	6.3 ± 0.6	44.2 ± 11.2	78.0 ± 18.1

*HT* hamstring tendon graft, *PT* bone-patellar tendon graft, *AG* allograft

### Description of test battery

All subjects were evaluated by the same examiner. Subjects were asked to wear comfortable clothing and their own athletic shoes. The test battery consisted of the following measures that were conducted in this order: jump-landing task (LESS), SLH, TLH, SH and isokinetic test. Before testing, the subjects completed a warm-up of 10-min stationary cycling. Following warm-up, the subjects performed the jump-landing task assessed with the LESS to identify potentially high-risk movement patterns [33]. Briefly, the subject stands on a 30-cm box with a target line drawn on the floor at a distance of half of the individual’s height. The subject was instructed to jump forward from the box and land just past the marked line with both feet landing simultaneously and immediately rebound by jumping for maximum vertical height [33]. Two standard 60-Hz video cameras (Sony; DSR-hc62, Tokyo, Japan) captured frontal plane and sagittal plane views of the jump landing [15]. Each jump was videotaped and scored at a later date using the LESS score form [33]. Subjects performed 3 trials of a jump-landing task before test session commenced. Trials were excluded and repeated if the subjects jumped vertically from the box or if they did not jump for maximal height upon landing [33].

After the jump-landing task, patients performed the SLH, TLH and SH as described in detail previously [17, 31]. Three practice trials were performed to familiarize the subjects with the hop tasks. The subjects rested for 30 s between each jump trial and 3–5 min between the various hop tests to prevent fatigue.

Muscular performance was tested with an isokinetic device (Biodex System 3; Biodex Medical Systems, Inc, Shirley, NY) to test both legs at a velocity of 60°/s, 180°/s and 300°/s with 5, 10, 10 maximal concentric repetitions for flexion and extension, respectively. The uninvolved side was always tested first. There was a standardized 1-min rest between the various speed trials and 5 min after the isokinetic evaluation.

Following the physical tests, subjects completed 2 patient questionnaires: the IKDC and the ACL-RSI. The IKDC is a knee-specific outcome measure pertinent to a variety of knee conditions for assessing symptoms, function and sports activity [20]. The ACL-RSI is a 12-item patient-reported outcome of emotions, confidence in performance and risk appraisal after ACLR. It can discriminate psychological differences between athletes who returned to sports and those who did not after ACLR [4].

### Data reduction

The jump-landing task movements were analysed according to the LESS that is a valid and reliable (ICC = 0.91) clinical movement-analysis tool that evaluates jump-landing characteristics [33]. The LESS primarily uses a dichotomous scoring rubric to identify obvious movement errors, such as limited knee flexion or excessive medial knee displacement. Therefore, a 1-point differential in the total LESS score can be associated with moderate to large differences in certain biomechanical variables [33]. A higher LESS score indicates a greater number of landing errors and consequently poorer jump-landing technique. The average LESS score from the 3 test trials was used for data analyses. The hop tests used in the current study all have good to excellent ICCs with respective values for the SLH (ICC = 0.97), TLH (ICC = 0.80–0.92) and the SH (ICC = 0.84–0.96) [22, 29].

The isokinetic device (Biodex System 3; Biodex Medical Systems, Inc, Shirley, NY) used in the current has been shown to be highly reliable (ICC 0.91–0.99) [38]. As per recommendation of the European Board of Sports Rehabilitation (EBSR) muscle strength was expressed as an LSI as well in absolute values [36]. Absolute values were normalized to bodyweight (Nm/kg) for isokinetic test at 60°/s. A threshold for isometric quadriceps strength after ACLR has been recommended as >3.0 Nm/kg [23]. Although isokinetic strength was tested, the aforementioned threshold is almost identical to the 2.8 Nm/kg obtained during isokinetic tests in patients who were cleared to RTS after ACLR [42]. In addition, the hamstrings/quadriceps ratio was determined at 300°/s, as high knee flexion/extension angular velocities reveal significant gender differences, whereas low speeds do not [18]. Female athletes who demonstrated the combination of decreased relative hamstrings and high relative quadriceps strength were shown to be at increased risk of ACL injury [30].

The IKDC has been shown to be responsive over time [21]. The 15th percentile from the normative data from uninjured individuals was chosen as the cut-off score representative of the normal variance [26]. Patients under the age of 18 were classified according to normative data for individuals 18–24 years of age. For the ACL-RSI, a cut-off score of 56 points at 4 months post-ACLR predicted RTS at 12 months with a sensitivity of 58 % and specificity of 83 % [4].

Passing of the RTS test battery was defined to meet all of the following criteria:

1. LSI > 90 % isokinetic quadriceps and hamstrings strength at 60°/s, 180°/s and 300°/s [36],
2. normalized isokinetic quadriceps strength >3.0 Nm/kg for the involved leg at 60°/s [23],
3. hamstrings/quadriceps (H/Q) ratio >55 % for females and >62.5 % for males for the involved leg at 300°/s [18],
4. LESS < 5 [32]
5. LSI > 90 % for all single-leg hop tasks [36]
6. ACL-RSI > 56 points [4] and
7. a IKDC score within 15th percentile of healthy gender–age-matched subjects [26].

### Statistical analysis

A power analysis (*G*Power, version 3.1.7) was used to calculate the required sample size. With an effect size of 0.30 (medium–large effect ANOVA) and an alpha of 0.05, 20 subjects were required to obtain a power of 0.80 [10]. In total, 28 subjects were included, resulting in a total power of 0.81. All data were normally distributed as analysed with SPSS version 20 (SPSS Inc, Chicago, IL).

## Results

**Fig. 1:**
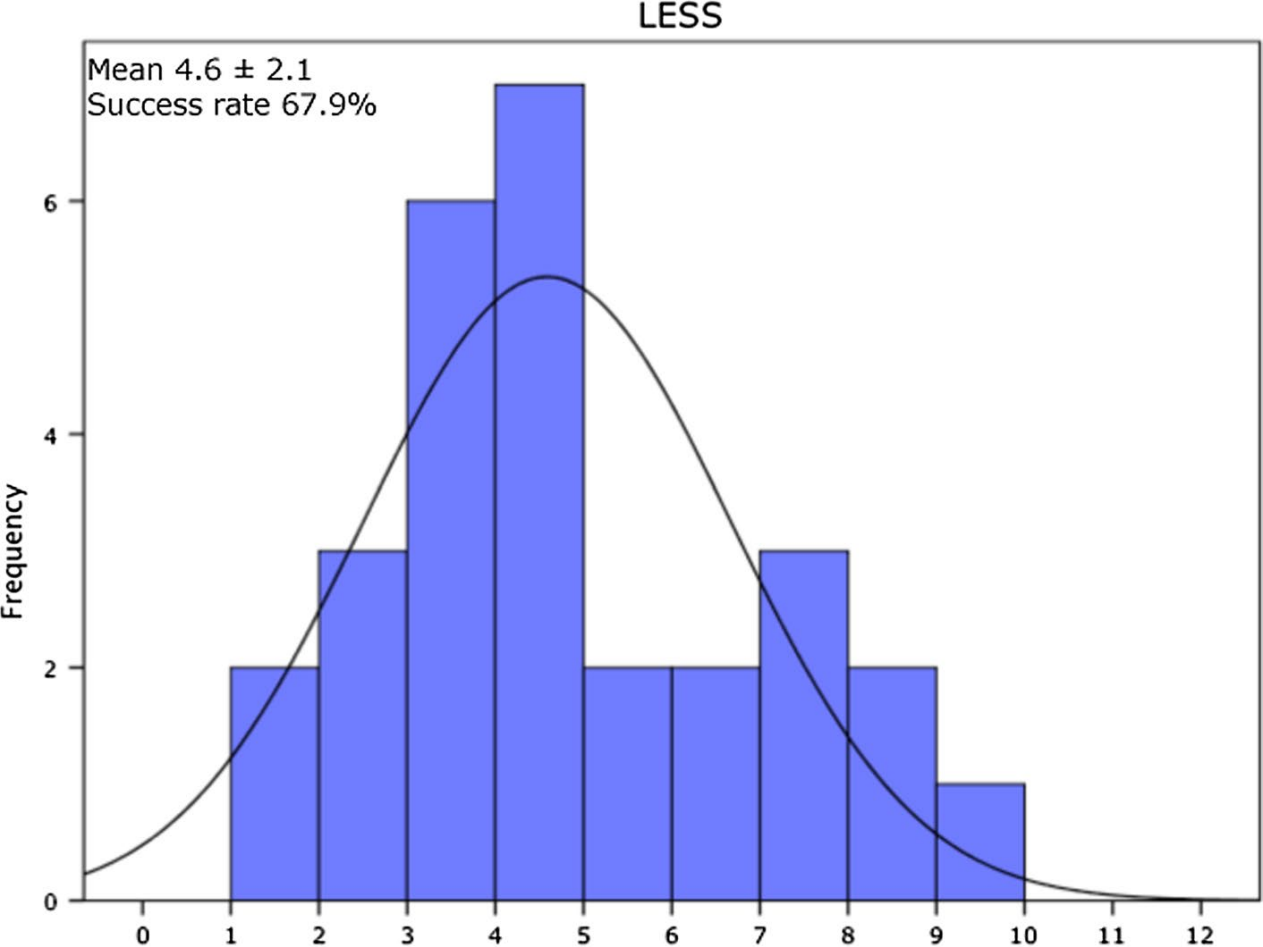
Results of the Landing error scoring system (LESS) presented as frequency distribution histogram. Mean and standard deviation (SD) of LESS and success rate (percentage of patients with a LESS 5 or lower) are also presented

**Fig. 2:**
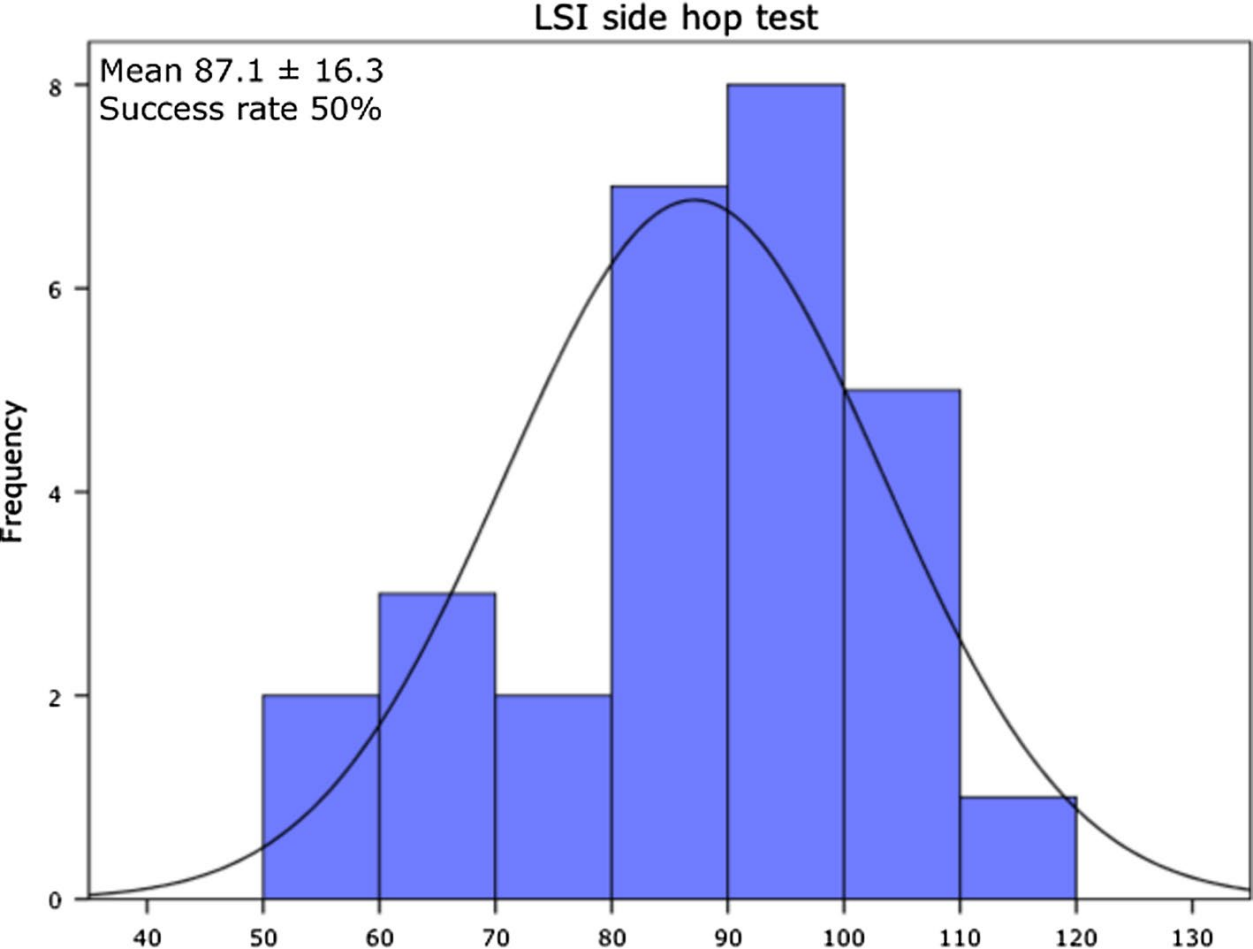
LSI peak for the side hop presented as frequency distribution histogram. Mean and standard deviation (SD) of LSI and success rate (percentage of patients with an LSI of 90 % or higher) are also presented

**Fig. 3:**
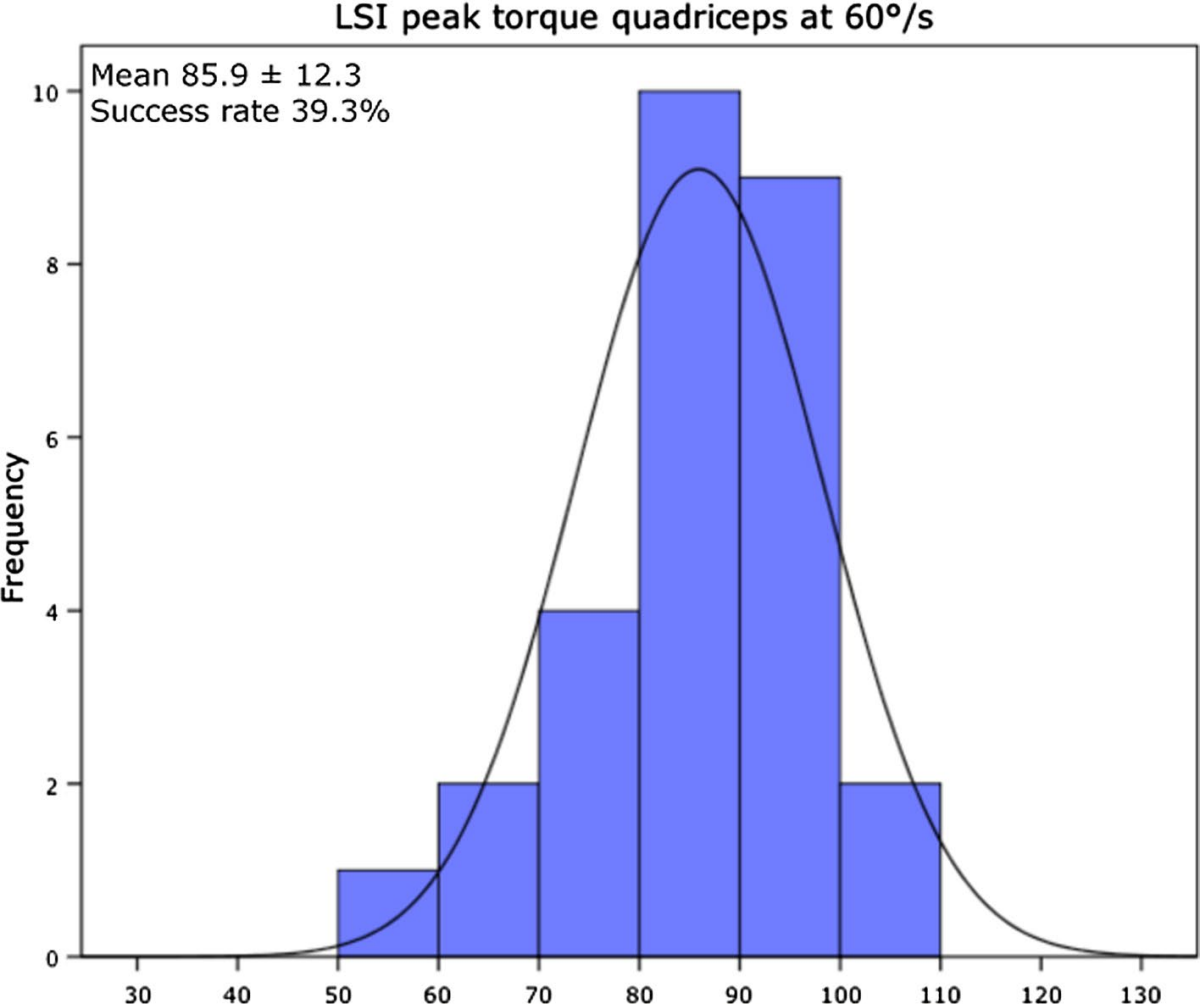
LSI peak torque quadriceps at 60°/s presented as frequency distribution histogram. Mean and standard deviation (SD) of LSI and success rate (percentage of patients with an LSI of 90 % or higher) are also presented

Two patients (7.1 %) passed all 11 criteria of the test protocol. In Table 2, the pass criteria are presented with the percentile of patients that passed the specific criterion. The pass criterion for the LESS < 5 was reached by 19 patients (67.9 %) of all patients. For the hop tests, 22 patients (78.5 %) of all patients passed LSI > 90.0 % for SLH, 24 patients (85.7 %) for TLH and 14 patients (50.0 %) for the SH. Eleven patients (39.3 %) out of 28 patients passed criteria for LSI peak torque quadriceps at 60°/s, 13 patients (46.4 %) at 180°/s and 12 patients (42.9 %) at 300°/s. For the hamstrings, 17 patients (60.7 %) passed LSI criteria at 60°/s, 15 (53.6 %) at 180°/s and 22 (78.6 %) at 300°/s, 78.6 % reached an LSI > 90. In total, 10 patients (35.7 %) of the patients passed criterion for the peak torque at 60°/s normalized to BW (>3.0 Nm/kg) for the involved leg. The H/Q ratio at 300°/s >55.0 % for females was achieved by 4 out of 6 female patients (66.7 %), and the >62.5 % criterion for males was achieved by 17 patients (75.3 %). Figures 1, 2 and 3 present the representative examples of the frequency distribution of individual LSI scores for LESS, LSI SH and LSI peak torque quadriceps at 60°/s. At 6 months post-ACLR, 24 patients (85.7 %) of the patients passed for the IKDC score (within 15 percentile of healthy gender–age-matched subjects), whereas 21 patients (75.0 %) had an ACL-RSI score >56.

**Table 2:** Pass criteria and percentile patients that passed the specific criterion

Pass criteria	Percentage of patients that passed criterion
LSI > 90 % peak torque quadriceps 60°/s	39.3
LSI > 90 % peak torque hamstrings 60°/s	60.7
LSI > 90 % peak torque quadriceps 180°/s	46.4
LSI > 90 % peak torque hamstrings 180°/s	53.6
LSI > 90 % peak torque quadriceps 300°/s	42.9
LSI > 90 % peak torque hamstrings 300°/s	78.6
Peak torque >3.0 Nm/kg for the involved limb 60°/s normalized to BW	35.7
H/Q ratio >55 % for females and >62.5 % for males for the involved limb at 300°/s	75.0
LSI > 90 % single-leg hop test	78.6
LSI > 90 % triple-leg hop test	85.7
LSI > 90 % side hop test	50.0
LESS < 5	67.9
IKDC score within 15 % of healthy gender–age-matched subjects	85.7
ACL-RSI > 56	75.0

## Discussion

The main findings from the current study were that patients that only 2 patients out of 28 passed the rigorous RTS battery. Our findings are in close agreement with those of others who used stringent criteria [37]. Thomee et al. [37] published a test battery in a prospective study that consisted of 6 tests, including 3 hop tests (vertical jump, hop for distance and side hop) and 3 strength tests (open-chain knee flexion, open-chain knee extension and closed-chain knee extension). At 6 months after ACLR, with success defined as those patients who scored LSI of >90 % in all 6 tests, none of the patients passed their criteria. In fact, only 23 % of all patients were successful at 2 years to reach the criteria.

The mean LESS score of 4.6 found in this study is lower compared to previous studies [8, 15, 24]. In the current study, the subjects frequently stated, although not analysed, they were somewhat apprehensive about the task. It is plausible the subjects focussed extensively on landing which could explain the relative good LESS scores. A more comprehensive analysis of the jump-landing task revealed that patients after ACLR had decreased knee flexion and increased valgus at initial contact as well as peak valgus. These findings are in agreement with work of others in that patients after ACLR demonstrate increased valgus [13, 24]. Goerger et al. [13] determined that alterations occur after ACL injury and ACLR, to both the involved and uninvolved leg, with an increase in hip adduction and knee valgus. For the ACL-injured leg, movement patterns are accompanied by a decrease in internal knee extension moment, internal hip flexion moment and anterior tibial shear force. These movement patterns found were identical to those shown to be predictive of ACL injury. A LESS score of 5 or more in elite-youth soccer athletes indicated they were at higher risk of sustaining ACL injuries compared to those with a lower LESS score [32]. Of note, 10 out of 28 patients had a LESS higher than 5. In a previous study, asymmetrical landing was also found (median LESS 6.5), with greater loading on the uninvolved leg after ACLR as a strategy to unload the involved leg [15]. These asymmetrical loading patterns are persistent after RTS and may be related to increased risk of second ACL injury [40]. However, it is currently not known what the clinical relevant cut-off score is for the LESS to identify patients at risk of second ACL injury.

Only 13 out of 28 patients (46.4 %) achieved LSI > 90 % for all 3 single-leg hop tasks. This reinforces the concept that hop tests in isolation may not be sensitive enough to capture deficits. Of interest, when more demanding testing was carried out that required increased stamina in the operative leg, as tested with the SH, results declined. This may indicate the profound effect of fatigue present in the involved extremity at the 6-month time period post-ACLR [1].

It may be important to relate a threshold of the LSI to the desired activity level: activities that are pivoting, contact or competitive and activities that are non-pivoting, non-contact or recreational [36]. In a study of 503 patients after ACLR, those patients with good hop test results (85 % LSI) for SLH and triple crossover were more likely to return than patients with poor results (LSI < 85 %) [5]. However, only 33 % of all patients attempted competitive sports at 1 year after ACLR [5].

For strength tests, the most important finding was that only 39.3 % of all patients passed the criterion for LSI peak torque quadriceps at 60°/s. This is in agreement with findings of a systematic review showing that 6–9 months post-ACLR, patients had significant lower muscle strength compared to the control group with differences in LSI between 16 and 39 % and were, therefore, not within the acceptable LSI limit [25]. The results show that not only do patients after ACLR exhibit side-to-side deficits, but the uninvolved leg of ACLR is also significantly weaker to a matched leg of a control group. This implies that the uninvolved leg is significantly affected by the ACL injury, questioning to use the LSI as a criterion for RTS [25]. The overall pattern is that the ACL-reconstructed leg is weaker than the uninvolved leg, which itself is weaker than that seen in matched healthy controls. Based on a review that included 39 studies, the reviewers concluded that isokinetic strength measures have not been validated as useful predictors of successful RTS [39]. Others also found weak evidence that supports an association between higher quadriceps strength and RTS [11]. A recently presented RTS test battery determined that quadriceps strength deficits prior to return to level I sport were a significant predictor of a knee reinjury [16]. Interestingly of the 74 patients who returned to level I sports, the 51 patients who did not sustain a second knee injury had a mean quadriceps LSI of 84.4 % [16] which is below the recommended LSI > 90 % [36].

The ACLR group in the current study had an average IKDC score of 80.8 that indicates that a majority were below the age-matched 15th percentile that is considered to be indicative of normal knee function [2]. In a well conducted systematic review, Czuppon et al. [11] found conflicting evidence for associations between RTS and the IKDC subjective form score, post-operative hamstring strength and LSI for single hop or crossover hop for distance. Relying solely on the IKDC as an indicator of normal knee function in regard to the ability to pass RTS criteria has been questioned [26]. Researchers found that patients who scored poorly on the IKDC were over 4 times more likely to fail the RTS tests. However, for the athletes who scored well on the IKDC, nearly 50 % overestimated their recovery. In other words, good IKDC scores did not necessarily mean the athletes would pass the RTS tests [26]. This indicates that the decision regarding RTS cannot be made based on the IKDC results alone.

The mean ACL-RSI score was 67.8, and 7 out of 28 patients did not meet the cut-off score of 56. Ardern et al. [4] reported that a ACL-RSI of 40.4 at 4 months post-ACLR was predictive for patients who did not return to pre-injury sports at 12 months after ACLR. In our patient sample, only 1 patient had a score below this threshold and obtained a score of 30.

The test battery presented has not been validated in terms of accuracy to predict second injury, and the patient sample size was small. However, the purpose of this paper was to initiate development of a new test battery as it is currently unknown what components of human movement like strength, endurance, balance alone and in combination is needed to achieve the optimal validity in regard to safe RTS. In line with the evidence supporting the multifactorial aetiology of ACL injuries, there is a need for a multifactorial RTS framework and the use of multivariate models in the future studies, to understand the complexity of RTS after ACLR [35]. The authors feel that the presented RTS test battery is a first step in that direction.

It needs to be clarified whether RTS is to a pivoting or non-pivoting sport, contact or non-contact sport, the same pre-injury sport, the same competitive level, the same sport but on a lower level, a different sport, or that the athlete merely perceives that the return to sport is successful [7, 36]. The use of the term RTS must be accompanied by a detailed description of the type and level of activity, as well as the time of return and duration of participation [3]. To enhance athlete’s chance to return to the same sport (at the same level) whilst minimizing reinjury risk, sport-specific tests should be incorporated in RTS tests for athletes after ACLR [9].

The test battery presented by the authors can be easily adopted by clinicians in day by work. The requirements for equipment are very minimal: space for hop tests, measurement tape, a 30-cm-high box, 2 video cameras and a hand-held dynamometer (instead of isokinetic device) and patient questionnaires. The performance-based and patient-reported outcomes can provide clinically relevant data throughout rehabilitation to identify deficits that subsequently can be targeted with interventions prior to safe release to sports.

## Conclusion

The evidence emerging from this study suggests that the majority of patients who are 6 months after ACLR require additional rehabilitation to pass RTS criteria. The RTS battery described in this study may serve as a framework for future studies to implement multivariate models in order to optimize the decision-making regarding RTS after ACLR with the aim to reduce incidence of second ACL injuries.
